# Schistosomicidal Activity of the Essential Oil of *Ageratum conyzoides* L. (Asteraceae) against Adult *Schistosoma mansoni* Worms

**DOI:** 10.3390/molecules16010762

**Published:** 2011-01-18

**Authors:** Nathalya I. de Melo, Lizandra G. Magalhaes, Carlos E. de Carvalho, Kamila A. L. Wakabayashi, Gabriela de P. Aguiar, Rafael C. Ramos, Andre L. L. Mantovani, Izabel C. C. Turatti, Vanderlei Rodrigues, Milton Groppo, Wilson R. Cunha, Rodrigo C. S. Veneziani, Antônio E. M. Crotti

**Affiliations:** 1 Núcleo de Pesquisas em Ciências Exatas e Tecnológicas, Universidade de Franca, CEP 14404-600, Franca, SP, Brazil; E-Mails: nathalyaisabel@gmail.com (N.I.M.); lizandraguidi@unifran.br (L.G.M.); denossauro7@gmail.com (C.E.C.); kamilakemi@hotmail.com (K.A.L.W); gaby.alp@hotmail.com (G.P.A); rafaelcorrearamos@hotmail.com (R.C.R.); a12sagitary@yahoo.com.br (A.L.L.M.); wrcunha@unifran.br (W.R.C.); rcsvenez@unifran.br (R.C.S.V.); 2 Faculdade de Ciências Farmacêuticas de Ribeirão Preto, Universidade de São Paulo, CEP 14040-903 Ribeirão Preto, SP, Brazil; E-Mail: izcristu@usp.br (I.C.C.T.); 3 Faculdade de Medicina de Ribeirão Preto, Universidade de São Paulo, CEP 14049-900 Ribeirão Preto, SP, Brazil; E-mail: vrodrigu@fmrp.usp.br (V.R.); 4 Faculdade de Filosofia, Ciências e Letras de Ribeirão Preto, Universidade de São Paulo, CEP 14040-901 Ribeirão Preto, SP, Brazil; E-mail: groppo@ffclrp.usp.br (M.G.)

**Keywords:** *Schistosoma*, essential oil, *Ageratum conyzoides*

## Abstract

The *in vitro* schistosomicidal effects of the essential oil of *Ageratum conyzoides* L. (Ac-EO) against adult worms of *Schistosoma mansoni* is reported in this paper. Concerning this activity, Ac-EO was considered to be active, but less effective than the positive control (praziquantel, PZQ) in terms of separation of coupled pairs, mortality, decrease in motor activity, and tegumental alterations. However, Ac-EO caused an interesting dose-dependent reduction in the number of eggs of *S. mansoni*. Precocene I (74.30%) and (*E*)-caryophyllene (14.23%) were identified as the two major constituents of Ac-EO. These compounds were tested individually and were found to be much less effective than Ac-EO and PZQ. A mixture of the two major compounds in a ratio similar to that found in the Ac-EO was also less effective than Ac-EO, thus revealing that there are no synergistic effects between these components. These results suggest that the essential oil of *A. conyzoides* is very promising for the development of new schistosomicidal agents.

## 1. Introduction

Schistosomiasis, a disease caused by trematode flatworms of the genus *Schistosoma*, is one of the most prevalent tropical diseases in the World [1]. Pointed out as a major neglected pathology, it is estimated that 200 million people are infected with this parasite worldwide, and that approximately 779 million are at risk of contracting it [2,3]. The disease burden exceeds 70 million disability-adjusted life years [4]. Its treatment is based on the control of adult worms in infected patients, being praziquantel (PZQ) the most widely used drug. Nevertheless, the long-term application of PZQ results in decreased efficiency and appearance of resistant strains [5,6,7]. Moreover, PZQ is often out of reach for the population living in developing countries [6]. The growing need for the development of novel and inexpensive drugs against schistosomiasis has led the scientific community to intensify the search for extracts and pure compounds obtained from plants exhibiting potential schistosomicidal properties [6,8]. However, only a few number of essential oils have been investigated for their schistosomicidal potential [9].

As part of our ongoing project on the anti-parasitary activity of essential oils [10], we report herein the *in vitro* schistosomicidal activity of the essential oil of *Ageratum conyzoides* L. (Asteraceae), an annual aromatic weed from Southeastern Brazil. This species is commonly found in tropical and subtropical zones and is popularly known as “mentrasto” in Portuguese [11,12]. It is employed in folk medicine as purgative, febrifuge, antiasthmatic, antispasmodic, analgesic, antidiarrhoeic, anti-inflammatory, against colic and for headache relief [13]. The essential oil of such species has been reported acting as insecticide [14,15], fungicide [16,17], anti-inflammatory [18], and antitumor [19] agent. Although the antihelmintic and nematicidal properties of this essential oil have also been reported [12], its effects on adult worms of the genus *Schistosoma*, on the egg laying capacity of this worm, and on egg development have not been investigated so far.

## 2. Results and Discussion

In the last years, several *in vitro* studies have been performed to search for new active compounds against *Schistosoma* species [3,20,21,22,23,24]. In this study, *in vitro* effects of different concentrations of the essential oil of *A. conyzoides* (Ac-EO) on *S. mansoni* adult worms were evaluated. As shown in Table 1, praziquantel (PZQ), which was used as positive control, resulted in the death of all the parasites within 24 h at a concentration of 10 μg/mL, whereas no mortality was observed in the worms belonging to the negative control groups (RPMI 1640 medium and DMSO 1% plus RPMI 1640 medium). On the other hand, the Ac-EO at 50 μg/mL caused the death of 50% of *S. mansoni* male and female adult worms after 24 h of incubation. However, incubation with the Ac-EO at 100 μg/mL, resulted in the death of most of the *S. mansoni* adult forms of (75% females and 100% males) after 120 h. The concentrations of the Ac-EO required to kill 50% (LC_50_) of the adult worms *in vitro* were calculated to be 198.8 and 75.70 μg/mL in the periods of 24 and 120 h, respectively. These results not only showed that the Ac-EO exhibited *in vitro* schistosomicidal activity, but also indicated that *S. mansoni* male worms are more susceptible to the Ac-EO than female ones. The differences between *S. mansoni* male and female worms in terms of susceptibility have also been reported in cases in which praziquantel [25] and ginger extract [26] were employed.

**Figure 1:**
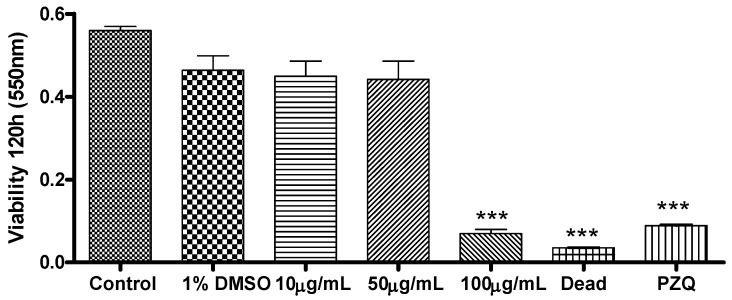
*In vitro* effects of Ac-EO on the viability of *S. mansoni* adult worms. Pairs of adult worms were treated with Ac-EO at different concentrations, for 120 h, and the viability was measured by MTT assay at 550 nm. RPMI 1640 medium and 1% DMSO + RPMI 1640 medium were used as negative controls. Praziquantel (PZQ, 10 μg/mL) and heat-killed worms at 56 °C were used as positive control groups. Data are presented as the mean of four experiments. *** P < 0.001.

The viability of the adult worms was also evaluated during their *in vitro* incubation with Ac-EO at 10, 50, and 100 μg/mL (Figure 1). In the case of the groups treated with Ac-EO at 10 and 50 µg/mL, the viability of the adult worms was similar to that of the negative control groups at 120 h of incubation. Surprisingly, the group of adult worms treated with Ac-EO at 100 µg/mL had significantly diminished viability compared with the negative control group, but this viability was similar to the one observed for the positive control (PZQ) group. These results revealed an interesting non-linear dose-response effect of Ac-EO at the tested concentrations and corroborated the microscopic analysis results.

When the *S. mansoni* adult worms were incubated with the Ac-EO at 100 μg/mL, there was a significant reduction in their motor activities after 24 h (Table 1). All the parasites belonging to the positive control group (PZQ) at 10 μg/mL also had total decreased motor activity within 24 h. In contrast, there were no tegumental changes in the worms of the groups incubated with the Ac-EO, even after 120 h, and the negative control. Otherwise, 75% of the worms treated with PZQ displayed tegumental alterations in the same period, in accordance to previous studies on PZQ [27].

In order to evaluate the *in vitro* effects of the Ac-EO on the reproductive fitness of *S. mansoni*, the ability of this oil to promote separation of the coupled adult worms into individual male and female, and to inhibit the oviposition was also investigated. As shown in Table 1, the Ac-EO promoted separation of 50% and 75% of the coupled pairs of worms after 120 h at concentrations of 50 and 100 μg/mL, respectively. On the other hand, the parasites incubated with the Ac-EO at 10 μg/mL and those belonging to the negative control groups (RPMI 1640 medium and DMSO 1% plus RPMI 1640 medium) remained coupled, even after 120 h. It has been reported that other plant constituents, such as curcumin, extracted from the rhizome of *Curcuma longa* [23], and piplartine, an amide isolated from *Piper tuberculatum* [24] have effects on the egg production. The Ac-EO at 50 μg/mL was observed to cause a slight decrease in the number of eggs when compared to the negative control (data not reported here). In this case, however, this effect could be a consequence of the separation of the coupled worms, which took place at that concentration and that precludes any reproductive process. Otherwise, the number of eggs in the group incubated with the Ac-EO at 10 μg/mL, in which the separation of the coupled pairs or worms has not occurred, was observed to be similar to that obtained in the group incubated with the negative control (data not included here). These results demonstrated that the Ac-EO can promote the separation of coupled worms but it has no significant effect on the number of eggs. 

**Figure 2:**
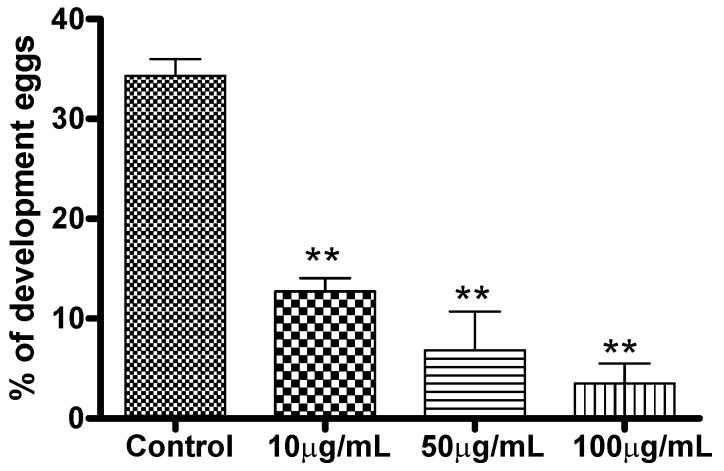
*In vitro* effects of the Ac-EO on egg development. Quantitative analysis of the development of the phenotype. After treatment, the eggs were microscopically examined and scored as developed or undeveloped on the basis of the presence or absence of the miracidium. Data are presented as the mean of developed eggs from three separate experiments. ** P < 0.01.

Although the Ac-EO had no effect on egg production, as previously discussed in this paper, it was observed that this oil significantly reduced the percentage of developed eggs in a dose-response dependent manner at 120 h of incubation (Figure 2). PZQ, the drug that is most widely used in the treatment of schistosomiasis was not tested because it is reported to be inactive against developing schistosomes [28].

**Figure 4:**
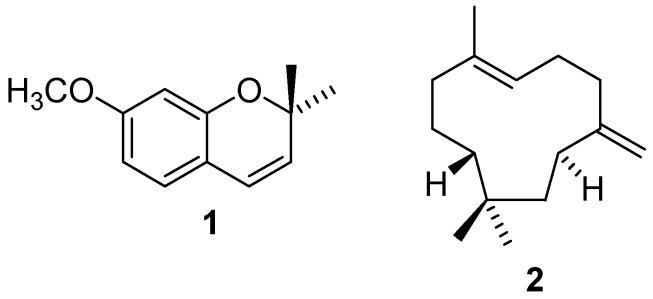
Chemical structures of precocene I (**1**) and (*E*)-caryophyllene (**2**).

The essential oil of the leaves of *A. conyzoides* (Ac-EO) was obtained in 0.09 % yield (w/w). GC-MS analysis revealed that precocene I (74.3%) and (*E*)-caryophyllene (14.23%) are the major components of such oil (Table 2). Its chemical composition was found to be similar to that reported by other authors [13,29,30,31,32]. Thus, in order to verify whether the schistosomicidal activity of EO-Ac is related, at least in part, to the presence of these two major compounds, precocene I (**1**) and (*E*)-caryophyllene (**2**) (Figure 4) were individually tested. As depicted in Table 3, both compounds caused no mortality or tegumental alterations in the adult worms, even at a concentration of 200 μM. Precocene I (200 μM) gave rise to a separation of 50% of the couple at 24 h of incubation, whereas worms remained coupled after incubation in (*E*)-caryophyllene, even at higher concentrations. Also, precocene I and (*E*)-caryophyllene at 100 μM slightly decreased the motor activity at 120 h of incubation. These data demonstrate that the Ac-EO was more active against *S. mansoni* adult worms than its major constituents (**1** and **2**) alone.

Finally, aiming to verify the occurrence of possible synergistic and/or additive effects between precocene I and (*E*)-caryophyllene in the Ac-EO, a solution of compounds **1** and **2** at a 4:1 (w/w) ratio, which is similar to that found in Ac-EO, was prepared and evaluated for its schistosomicidal activity, as previously described in this paper. Comparison between data from Table 3 and Table 1 evidence that the mixture of **1** and **2** did not exhibit any *in vitro* schistosomicidal activity against *S. mansoni*, except for a slight reduction in the motor activity at 100 μg/mL and at 24 h of incubation. These results suggest that the *in vitro* schistosomicidal activity of Ac-EO may be related to minor constituents present in the essential oil or ruled by more intricate synergistic and/or additive relationships.

## 3. Experimental

### 3.1. Plant material

*Ageratum conyzoides* L. (Asteraceae) was collected at “Sítio 13 de maio” near Franca city (20°26’S 47°27’W 977 m, State of São Paulo, Brazil) in May 2010. A voucher specimen (SPFR10014) has been deposited at the Herbarium of Departamento de Biologia, Faculdade de Filosofia, Ciências e Letras de Ribeirão Preto, Universidade de São Paulo, São Paulo, Brazil. (Herbarium SPFR).

### 3.2. Chemicals

The essential oil of *A. conyzoides* was obtained from fresh leaves by hydrodistillation in a Clevenger-type apparatus for 3 h. After manual collection of the essential oil, traces of water were removed by freezing the sample below 0 °C, followed by transfer of the unfrozen essential oil to a new vial. Yield was calculated from the weight of fresh leaves. Precocene I and (*E*)-caryophyllene were purchased from Sigma-Aldrich Co. (St. Louis, MO, USA).

### 3.3. GC-MS analysis

The essential oil of *A. conyzoides* obtained by hydrodistillation was analyzed by GC-MS on a Shimadzu QP2010 Plus (Shimadzu Corporation, Kyoto, Japan) system equipped with a AOC-20i autosampler under the following conditions: Restek Rtx-5MS fused silica capillary column (30 m × 0.25 mm i.d. × 0.25 μm film thickness), composed of 5%-phenyl-95%-methylpolysiloxane operating in the electron ionization mode at 70 eV. Helium (99.999%) was used as the carrier gas at a constant flow of 1.0 mL/min. The injection volume was 0.1 μL (split ratio of 1:10), the injector temperature was 240 °C, and the ion-source temperature was 280 °C. The oven temperature was programmed to increase from 60 °C to 240 °C at 3 °C/min. Mass spectra were taken with a scan interval of 0.5 s and mass range from 40 to 600 Da. Quantification of each constituent was estimated by internal normalization (%). Identification of the Ac-EO components was based on their retention indices, relative to a homologous series of *n*-alkanes (C_8_–C_20_), on an Rtx-5MS capillary column under the same operating conditions and computer matching with the Wiley 7, NIST 08 and FFNSC 1.2 spectra libraries, as well as by comparison of their mass spectra with those reported in the literature.

### 3.3. Parasite culture and maintenance

The LE strain of *S. mansoni* was maintained by passage through *Biomphalaria glabrata* snails and Balb/c mice. After 8 weeks, *S. mansoni* adult worms (pairs) were recovered under aseptic conditions from mice previously infected with 200 cercariae by perfusion of the livers and mesenteric veins [33]. The worms were washed in Roswell Park Memorial Institute (RPMI) 1640 medium (Invitrogen), kept at pH 7.5 with HEPES 20 mM, and supplemented with penicillin (100 UI/mL), streptomycin (100 μg/mL), and 10% bovine fetal serum (Gibco). After washing, one pair of adult worms was transferred to each well of a 24-well culture plate containing 2 mL of the same medium and incubated at 37 °C in a humid atmosphere containing 5% CO_2_ prior to use. At 24 h after incubation, the essential oil from the leaves of *Ageratum conyzoides* (Ac-EO) or its major compounds were dissolved in 1% dimethyl sulfoxide (DMSO) and added to RPMI 1640 medium. The effects of Ac-EO on *S. mansoni* were assessed by observing the worms’ viability, as well as pairing, egg production, and egg development. All experiments were authorized by the Ethical Committee for Animal Care of the University of São Paulo and University of Franca, and were in accordance with the national and international accepted principles for laboratory animal use and care.

### 3.4. In vitro studies with Schistosoma mansoni

For the *in vitro* test with *S. mansoni*, Ac-EO and the pure compounds were dissolved in 1% DMSO and used at concentrations of 10, 50, and 100 μg/mL for Ac-EO, and of 25, 50, 100, and 200 μM for the pure compounds, which were added to the medium containing one adult worm pair after a period of 24 h of adaptation to the culture medium. The parasites were monitored at 24 h and 120 h, to evaluate their general condition: motor activity, alterations in the tegument, and mortality rate [34]. Also, changes in the pairing, egg production, and egg development were examined by using an inverted microscope (Leitz) [23,35]. The control worms were treated with 1% DMSO in RPMI 1640 medium. Four replicates of all experiments were carried out using RPMI 1640 medium and RPMI 1640 with 1% DMSO as negative control groups. Praziquantel (PZQ) was used as positive control group at concentrations of 10 μg/mL in the assays with Ac-EO and the mixture of precocene I and (E)-caryophyllene (4:1 w/w), and 10 μM in the assays with the pure compounds. The LC_50_ was calculated from dose-response inhibition graph [24].

### 3.5. Viability assay

Pairs of adult worms were incubated for 24 or 120 h with Ac-EO (10, 50, or 100 μg/mL), and the viability assay was performed by means of the MTT assay [36]. After incubation, each pair of adult worms was placed individually into wells (96-well plates) containing phosphate-buffered saline (100 μL) with 5 mg MTT per milliliter for 30 min, at 37 °C. The solution was carefully removed and replaced with DMSO (200 μL), and the worms were allowed to stand in DMSO at room temperature for 1 h. The absorbance was read at 550 nm using an ELISA reader (Tecan A-5082, Salzburg, Austria). Parasites in RPMI 1640 medium and RPMI 1640 with 1% DMSO were used as negative control groups and Heat-killed worms at 56 °C and 10 μM PZQ were used as positive control groups. Four replicates of all experiments were accomplished.

### 3.6. Statistical analysis

Results are expressed as mean ± SEM. Data were statistically analyzed by one-way analysis of variance, followed by Tukey’s multiple comparison test.

## 4. Conclusions

In summary, we have reported herein an investigation of the *in vitro* schistosomicidal potential of the essential oil of *A. conyzoides* and its major constituents for the first time. We have concluded that such oil exhibits *in vitro* schistosomicidal activity against *S. mansoni* adult worms, although they are less effective than PZQ with respect to the separation of coupled pairs, mortality, decrease in motor activity, and tegumental alterations. Also, our results have demonstrated that Ac-EO prompts an interesting reduction in the number of developed eggs in a dose-dependent manner. This is a remarkable finding, since the drug that is most widely employed for the treatment of this disease is known to be active only against the adult forms of the parasite. In this context, the *in vitro* schistosomicidal effects of the essential oil of *A. conyzoides* reported herein indicate that it could be considered a promising source for the development of new schistosomicidal agents. Further biological studies to elucidate its mechanism(s) of its schistosomicidal action are already in progress in our laboratories.

## Figures and Tables

**Table 1:** *In vitro* effects of Ac-EO against *S. mansoni* adult worms.

Group	Incubation period (h)	Number of separated worms (%)	Number of dead worms (%)		Decrease in motor activity				Number of worms with tegumental alteration			
					Slight (%)		Significant (%)		Partial (%)		Extensive (%)	
			M	F	M	F	M	F	M	F	M	F
Control^a^	24	0	0	0	0	0	0	0	0	0	0	0
	120	0	0	0	0	0	0	0	0	0	0	0
DMSO 1%^b^	24	0	0	0	0	0	0	0	0	0	0	0
	120	0	0	0	0	0	0	0	0	0	0	0
PZQ^c^	24	0	100	100	0	0	100	100	25	25	50	50
10 μg/mL	24	0	0	0	0	0	0	0	0	0	0	0
	120	0	0	0	25	25	0	0	0	0	0	0
50 μg/mL	24	50	0	0	25	25	0	0	0	0	0	0
	120	50	25	0	50	50	0	0	0	0	0	0
100 μg/mL	24	50	25	0	0	0	100	100	0	0	0	0
	120	75	100	75	0	0	100	100	0	0	0	0

^a,b^ Negative control groups (^a^ RPMI 1640; ^b^ DMSO + RPMI medium); ^c^ Positive control at 10 μg/mL. **M**: males; **F**: females.

**Table 2:** Chemical constituents of the essential oil of *A. conyzoides* identified by GC-MS.

Compound	Retention time (min)	Retention index	Peak area (%)
α-Thujene	4.909	924	0.16
α-Pinene	5.100	931	0.30
β-Cubebene	22.312	1384	0.62
β-Elemene	22.396	1386	0.42
(*E*)-Caryophyllene	23.594	1414	14.23
α-Humulene	25.059	1450	2.80
Precocene I	25.517	1461	74.30
γ-Muurolene	26.129	1476	3.44
Germacrene D	26.546	1486	0.59
Bicyclogermacrene	26.702	1490	3.14
**Total**			**100.00**

**Table 3:** *In vitro* effects of Precocene I (**1**), (*E*)-caryophyllene (**2**), and the **1 + 2** (4:1 w/w) mixture against *S. mansoni* adult worms.

Group	Incubation period (h)	Number of separated worms (%)	Number of dead worms (%)		Decrease in motor activity				Number of worms with tegumental alteration			
					Slight (%)		Significant (%)		Partial (%)		Extensive (%)	
			M	F	M	F	M	F	M	F	M	F
Control^a^	24	0	0	0	0	0	0	0	0	0	0	0
	120	0	0	0	0	0	0	0	0	0	0	0
PZQ ^b^ 10 μM	24	0	100	100	0	0	100	100	25	25	50	50
(3.1 μg/mL)												
(**1**) 25 μM	24	0	0	0	25	25	0	0	0	0	0	0
(4.7 mg/mL)	120	0	0	0	25	25	0	0	0	0	0	0
(**1**) 50 μM	24	0	0	0	25	0	0	0	0	0	0	0
(9.4 mg/mL)	120	0	0	0	50	50	0	0	0	0	0	0
(**1**) 100 μM	24	0	0	0	25	25	0	0	0	0	0	0
(18.8 mg/mL)	120	0	0	0	75	100	0	0	0	0	0	0
(**1**) 200 μM	24	50	0	0	75	75	0	0	0	0	0	0
(37.6 mg/mL)	120	50	0	0	100	100	0	0	0	0	0	0
(**2**) 25 μM	24	0	0	0	25	25	0	0	0	0	0	0
(5.2 mg/mL)	120	0	0	0	25	25	0	0	0	0	0	0
(**2**) 50 μM	24	0	0	0	25	25	0	0	0	0	0	0
(10.4 mg/mL)	120	0	0	0	50	50	0	0	0	0	0	0
(**2**) 100 μM	24	0	0	0	50	75	0	0	0	0	0	0
(20.8 mg/mL)	120	0	0	0	100	100	0	0	0	0	0	0
(**2**) 200 μM	24	0	0	0	75	0	0	0	0	0	0	0
(41.6 mg/mL)	120	0	0	0	100	100	0	0	0	0	0	0
(**1**+**2**) 25 μg/mL	24	0	0	0	25	25	0	0	0	0	0	0
	120	0	0	0	25	25	0	0	0	0	0	0
(**1**+**2**) 50 μg/mL	24	0	0	0	50	50	0	0	0	0	0	0
	120	0	0	0	50	50	0	0	0	0	0	0
(**1**+**2**) 100 μg/mL	24	0	0	0	100	100	0	0	0	0	0	0
	120	0	0	0	100	100	0	0	0	0	0	0
(**1**+**2**) 200 μg/mL	24	0	0	0	100	100	0	0	0	0	0	0
	120	0	0	0	100	100	0	0	0	0	0	0

^a^ RPMI 1640. **M**: males; **F**: females.
